# Control of breathing in preterm infants on incubator oxygen or nasal cannula oxygen

**DOI:** 10.1038/s41390-024-03460-5

**Published:** 2024-08-15

**Authors:** Colm P. Travers, Rouba Chahine, Arie Nakhmani, Inmaculada Aban, Waldemar A. Carlo, Namasivayam Ambalavanan

**Affiliations:** 1 Department of Pediatrics, University of Alabama at Birmingham, Birmingham, AL USA; 2 Social, Statistical and Environmental Sciences Unit, RTI International, Research Triangle Park, NC USA; 3 Department of Biostatistics, University of Alabama at Birmingham, Birmingham, AL USA; 4 Department of Electrical and Computer Engineering, University of Alabama at Birmingham, Birmingham, AL USA

## Abstract

**Background:**

Incubator oxygen may improve respiratory stability in preterm infants compared with nasal cannula oxygen.

**Methods:**

Single center randomized trial of infants <29 weeks’ gestation on supplemental oxygen at ≥32 weeks’ postmenstrual age. Infants were crossed-over every 24 hours for 96 hours between incubator oxygen and nasal cannula ≤1.0 L/kg/min. We measured episodes of intermittent hypoxemia (oxygen saturations (SpO_2_) < 85% ≥10 seconds), bradycardia, cerebral and abdominal hypoxemia, and end-tidal carbon dioxide.

**Results:**

We enrolled 25 infants with a gestational age of 26 weeks 4 days±15 days (mean ± SD) and birth weight 805 ± 202 grams. There were no differences in episodes of intermittent hypoxemia, bradycardia, or cerebral hypoxemia between groups. There were fewer episodes of abdominal hypoxemia <40% ≥10 seconds with incubator oxygen compared with nasal cannula (132 ± 130 versus 158 ± 125; *p* < 0.01). Time with SpO_2_ < 85% and abdominal hypoxemia was lower among infants on incubator oxygen. Carbon dioxide values were higher while on incubator oxygen (41 ± 11 versus 36 ± 10 mmHg; *p* < 0.02).

**Conclusion:**

There was no difference in intermittent hypoxemia between incubator and nasal cannula oxygen among preterm infants on supplemental oxygen. Infants had higher levels of carbon dioxide while on incubator oxygen, which may have improved some measures of respiratory stability.

**ClincalTrials.gov identifiers:**

NCT03333174 and NCT03174301.

**Impact Statement:**

- In this randomized cross-over trial of preterm infants on supplemental oxygen, incubator oxygen did not decrease episodes of intermittent hypoxemia compared with nasal cannula oxygen. Incubator oxygen reduced time with oxygen saturations less than 85%, reduced abdominal hypoxemia, and increased carbon dioxide levels.
- Differences in measures of respiratory stability on incubator oxygen may be partly due to higher carbon dioxide levels compared with nasal cannula oxygen.
- The mode of supplemental oxygen administration may impact control of breathing in preterm infants through its effect on hypopharyngeal oxygen stability and carbon dioxide levels.

## Introduction

Immature control of breathing in preterm infants leads to recurrent apnea, hypopnea, and tachypnea, resulting in multiple episodes of hypoxemia and hyperoxemia.^1^ Intermittent hypoxemia has been associated with bronchopulmonary dysplasia, pulmonary hypertension, retinopathy of prematurity, and neurodevelopmental impairment in preterm infants.^2–5^ Conversely, hyperoxemia which frequently occurs due to efforts to prevent hypoxemia, is associated with increased retinopathy of prematurity.^6^ Even relatively small differences in achieved oxygen saturations (SpO_2_) can impact major outcomes among preterm infants including mortality and necrotizing enterocolitis.^7^ Despite increased awareness of the impact of monitoring oxygen saturation histograms, improved targeting remains a clinically relevant problem in the neonatal intensive care unit.^8^

Various methods of administering supplemental oxygen have been used to improve oxygen targeting.^9^ Closed loop automated oxygen control improves SpO_2_ targeting in infants on invasive and non-invasive ventilator support and high-flow nasal cannulae but closed-loop automated oxygen control requires equipment not available to most clinicians even in high-income countries and its effects on major outcomes are still under investigation.^10–13^ Incubator oxygen that is servo-controlled to a set fraction of inspired oxygen (FiO_2_) may reduce intermittent hypoxemia as it maintains a more stable pharyngeal effective FiO_2_ compared with low-flow nasal cannula oxygen.^1^ However, the effect on additional measures of oxygenation such as tissue oxygenation may provide greater insight into oxygen delivery, consumption, and the impact of mode of respiratory support.^14^ In addition, carbon dioxide levels may have an important impact on control of breathing but the effect of different modes of oxygen administration on carbon dioxide is not known.^15^ This study tested the hypothesis that among preterm infants <29 weeks’ gestation on supplemental oxygen at 32 weeks’ postmenstrual age, incubator oxygen would improve respiratory stability compared with a low-flow nasal cannula.

## Methods

This study was conducted at the Level IV Regional Neonatal Intensive Care Unit at the University of Alabama at Birmingham. The Institutional Review Board at the University of Alabama at Birmingham approved the study, which is registered with www.clinicaltrials.gov (NCT03333174). We screened preterm infants 22 weeks and 0 days to 28 weeks and 6 days of gestational age at birth with a birth weight from 400 to 1000 grams who were enrolled in the multicenter Pre-Vent Apnea observational study (Clinicaltrials.gov identifier: NCT03174301). We included infants at 32 weeks’ postmenstrual age if they were receiving supplemental oxygen either via a nasal cannula with flow rates ≤ 1.0 liter per kilogram per minute or a digitally-set servo-controlled incubator oxygen.^1^ Infants were enrolled from April 2018 to July 2021 if the parents/legal guardians consented. We excluded infants with a major congenital malformation, infants whose parents had withdrawn or refused consent, and infants deemed clinically unstable by the attending neonatologist.

### Recruitment and Randomization

Computer-generated block randomization sizes of 2 to 4 and sequentially numbered sealed opaque envelopes were used to achieve balance and reduce bias. Infants were randomized to two different intervention sequences (either cannula-incubator-cannula or incubator-cannula-incubator) using a 1:1 parallel allocation.

### Interventions

After randomization, infants were maintained for the first 24 hours on their baseline mode of supplemental oxygen therapy while attached to study monitors (Philips IntelliVue MP50 or MP70) that recorded SpO_2_, heart rate (HR), respiratory rate, and cerebral and abdominal (left lower quadrant) tissue saturations on near infrared spectroscopy (NIRS) (INVOS^TM^ 5100 C Cerebral/Somatic Oximeters) using infant/neonatal sensors (Medtronic, Dublin, Ireland). End-tidal carbon dioxide (EtCO_2_) was measured using an additional nasal cannula (Medtronic, Dublin, Ireland). TcCO_2_ was measured using the SenTec Digital Monitoring System (SenTec Inc, Fenton, MO) in three infants without EtCO_2_. We collected real-time numeric data at 1 Hz from patient monitors using ixTrend (ixitos GmbH, Berlin, Germany). We used uniform target SpO_2_ ranges of 91-95% for the duration of the study.^6^

Infants were then crossed-over randomly every 24 hours for an additional 72 hours to either intervention: cannula-incubator-cannula or incubator-cannula-incubator. When switching between modes of oxygen delivery, we calculated the effective FiO_2_ using standardized charts that account for weight, set FiO_2_, and flow.^16^ We used a 15–30 minute washout period after swapping between intervention modes before beginning recordings. Infants enrolled in the study received routine care from staff and remained on our central alarm system. Masking of the study intervention at the bedside was not done due to the multitude of clinically indicated adjustments in FiO_2_. Individuals analyzing the data were masked to the interventions.

### Measures

Our primary outcome was the number of intermittent hypoxemia (SpO_2_ < 85% for ≥ 10 seconds) episodes per 24 hours. Secondary outcomes were severe intermittent hypoxemia (defined as SpO_2_ < 80% for ≥ 10 seconds) episodes per 24 hours, the proportion of time with SpO_2_ < 85%, the proportion of time with SpO_2_ < 80%, the proportion of time with SpO_2_ > 95%, bradycardia (HR < 100 beats per minute for ≥ 10 seconds) episodes per 24 hours, episodes of cerebral (<55% for ≥ 10 seconds) and abdominal hypoxemia (<40% for ≥ 10 seconds) per 24 hours on near-infrared spectroscopy, and PCO_2_. Data were not imputed.

### Statistical analysis

We estimated that 25 infants were required to detect a 20% decrease in intermittent hypoxemia episodes while in incubator compared with nasal cannula oxygen, with β = 0.80 and two-tailed α = 0.05, with standard deviation at 0.5 of the mean. Results were analyzed by intention to treat. We used MATLAB (MathWorks, Natick, Massachusetts) to analyze numeric data for each 24 hour intervention period and SAS version 9.4 (Cary, NC) for all statistical analyses. We used generalized linear mixed models with random intercept to account for repeated measurements for the same subject over time. We used gamma distribution for continuous outcomes, negative binomial for count outcomes, and beta for proportions. The main goal was to test for treatment effect, given the washout period between treatment, we assumed that there was no carryover effect and did not include an interaction term in the model.^1^ A *p value* < 0.05 was considered statistically significant.

## Results

**Fig. 1:**
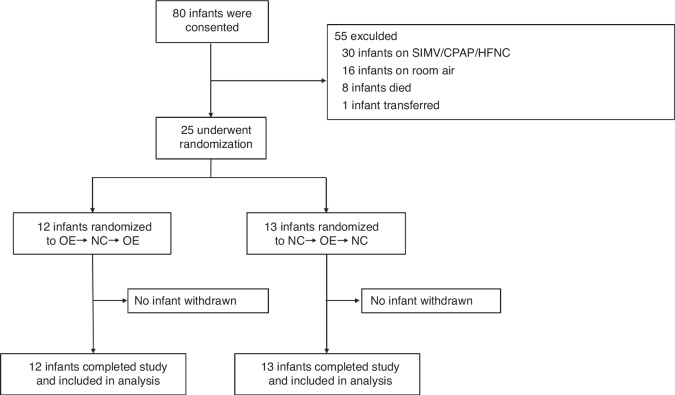
Flow diagram of study participants.

Twenty-five infants who met all inclusion/exclusion criteria were randomized (Fig. 1). They had a gestational age of 26 weeks 4 days ± 15 days (mean ± standard deviation) and birth weight 805 ± 202 grams. The postmenstrual age at study entry was 33 weeks 0 days ± 12 days (mean ± standard deviation) (Table 1). All but one infant met criteria for bronchopulmonary dysplasia at 36 weeks’ postmenstrual age. All infants completed the study and no infants were excluded from the analysis. Three infants did not complete the final day of testing because there were concerns for nasal irritation related to the use of the additional nasal cannula, two in the incubator first group and one in the cannula first group, leaving 72 days for analyses.

**Table 1:** Baseline Clinical characteristics of the 25 study participants.

Bronchopulmonary dysplasia at 36 weeks PMA, no. (%)	24 (96)
Gestational age, weeks and days ± days (mean ± SD)	26 4/7 ± 15/7
Birth weight, grams (mean ± SD)	805 ± 202
Postmenstrual age, weeks and days ± days (mean ± SD)	33 0/7 ± 12/7
Male, no. (%)	14 (56)
Race	
White, no. (%)	19 (76)
Black, no. (%)	6 (24)
Antenatal steroids, no. (%)	24 (96)
Surfactant, no. (%)	18 (72)
Postnatal steroids, no. (%)	2 (8)
Days ventilated before study entry, days (mean ± SD)	9 ± 11
Days on respiratory support, days (mean ± SD)	29 ± 21
Bronchopulmonary dysplasia at 36 weeks PMA, no. (%)	24 (96)

*SD* standard deviation.

*PMA* postmenstrual age.

Based on the repeated measure models, there were no significant differences in intermittent hypoxemia (96 ± 61 with incubator versus 106 ± 68 episodes/24 h with nasal cannula; *p* = 0.38). In addition, rates of severe intermittent hypoxemia, cerebral hypoxemia, or bradycardia did not differ significantly between incubator and nasal-cannula oxygen treatments (Table 2). There were fewer episodes of abdominal hypoxemia with incubator oxygen compared with nasal cannula oxygen (132 ± 130 with incubator versus 158 ± 125 with nasal cannula; *p* < 0.01).

**Table 2:** Outcomes by oxygen treatment type.

	Incubator Oxygen	Nasal Cannula	*p* value
Episodes of IH per 24 hours, mean ± SD	96 ± 61	106 ± 68	0.38^b^
Percent time SpO_2_ less than 85%, mean ± SD	4.2 ± 2.5	5.3 ± 3.6	0.04^c^
Episodes of severe IH per 24 hours, mean ± SD	36 ± 32	34 ± 26	0.83^b^
Percent time SpO_2_ less than 80%, mean ± SD	1.5 ± 1.3	1.6 ± 1.3	0.46^c^
Percent of time SpO_2_ less than 90%, mean ± SD	15.5 ± 5.8	17.9 ± 6.5	0.17
Percent time SpO_2_ more than 95%, mean ± SD	41.6 ± 14.0	37.7 ± 14.8	0.39^c^
Episodes of cerebral hypoxemia per 24 hours, mean ± SD	163 ± 164	162 ± 182	0.16^b^
Coefficient of variation of SpO_2_, mean ± SD	5.0 ± 1	4.7 ± 1.0	0.28^a^
Percent time cerebral NIRS < 55%, mean ± SD	20.6 ± 27.4	24.5 ± 32.3	0.22^c^
Episodes of abdominal hypoxemia per 24 hours, mean ± SD	132 ± 130	158 ± 125	<0.01^b^
Percent time abdominal NIRS < 40%, mean ± SD	16.4 ± 20.6	17.2 ± 20.5	0.04^c^
Episodes of bradycardia per 24 hours, mean ± SD	5 ± 7	4 ± 5	0.28^b^
Mean PCO_2_* (mmHg), mean ± SD	41 ± 11	36 ± 10	0.02^a^
Effective FiO_2_ during study, mean ± SD	0.27 ± 0.07	0.30 ± 0.07	0.03^a^

^a^*p*-value based on random effects mixed model based on gamma distribution.

^b^*p*-value based on random effects mixed model based on negative binomial distribution.

^c^*p*-value based on random effects mixed model based on beta distribution.

^*^Transcutaneous CO_2_ was measured in three infants without End-tidal CO_2_.

IH intermittent hypoxemia.

SpO_2_ oxygen saturations.

NIRS near infrared spectroscopy.

PCO_2_ carbon dioxide.

FiO_2_ fraction of inspired oxygen.

SD standard deviation.

Infants were exposed to a lower effective FiO2 during incubator oxygen compared with nasal cannula treatment (incubator vs. cannula: 0.27 ± 0.07 vs. 0.30 ± 0.07). The proportion of time with SpO_2_ < 85% and abdominal hypoxemia were lower during incubator oxygen compared with low-flow nasal cannula oxygen (Table 2). The proportion of time with SpO_2_ > 95%, SpO_2_ < 90%, SpO_2_ < 80%, or cerebral hypoxemia, and the coefficient of variation of oxygen saturations did not differ by treatment. PCO_2_ values were significantly higher while on incubator oxygen compared with low-flow nasal cannula (41 ± 11 mmHg vs. 36 ± 10 mmHg; *p* = 0.02).

## Discussion

In this randomized cross-over trial there was no difference in intermittent hypoxemia or bradycardia between incubator and nasal cannula oxygen among preterm infants on supplemental oxygen. Infants had higher levels of PCO_2_ while on incubator oxygen treatment, associated with improvement in some measures of respiratory stability including the percentage of time with oxygen saturations less than 85% and abdominal hypoxemia on near-infrared spectroscopy.

Our study included 25 extremely preterm infants on supplemental oxygen therapy and evolving bronchopulmonary dysplasia. No masking was possible so caregivers were aware of the mode of oxygen supplementation. It is possible that infants may have been treated differently by staff while receiving one treatment compared with the other. We masked our outcome assessors to study allocation and used mathematical algorithms to analyze electronically captured data recorded directly from patient monitors to reduce outcome ascertainment bias. It is possible that use of the additional nasal cannula to monitor end-tidal carbon dioxide may have inadvertently increased dead space and inspiratory and expiratory resistances in both groups and affected the results of this trial but no infant developed hypercapnia. The relative difference in episodes of intermittent hypoxemia was similar between our study and our previous study comparing incubator oxygen and nasal cannula oxygen.^1^ However, the current study had less statistical power than the previous study to detect a significant difference between groups.

In our previous trial, we crossed over 25 preterm infants systematically between nasal cannula and digitally-set servo-controlled incubator oxygen.^1^ Incubator oxygen was superior to nasal cannula oxygen for reducing hypoxemia but measures such as PCO_2_ and somatic oxygenation were not included. Carbon dioxide levels have important effects on control of breathing in preterm infants.^15,17,18^ Supplemental carbon dioxide decreases episodes of apnea in preterm infants.^19^ In the current study, carbon dioxide levels were higher during incubator oxygen treatment perhaps due to some washout effect with nasal cannula. It is possible that higher levels of carbon dioxide improved some measures of control of breathing during incubator oxygen therapy as noted in the aforementioned ECO study.^1^ However, there remained no difference in episodes of bradycardia between groups in our study in spite of the difference in carbon dioxide levels.^20^

In the current study, the proportion of time with abdominal hypoxemia and the number of episodes of abdominal hypoxemia was lower with incubator oxygen but there was no difference in cerebral hypoxemia by treatment. It is not clear why there was a difference in abdominal hypoxemia between treatments in our study. It is interesting to note that there were more episodes of cerebral and abdominal hypoxemia per day, as compared with intermittent hypoxemia episodes. The clinical significance and long-term implications of such frequent occult cerebral and abdominal desaturations need further study, as monitoring using routine pulse oximetry would not identify such events.^14^ The depth and duration of hypoxemia episodes may be important clinically.^3,5,21^ While desaturations less than 85% and 90% are associated with adverse outcomes in preterm infants, episodes less than 80%, and in particular those of longer durations, may be associated with an even higher risk of severe adverse outcomes.^3,5,6,21^ Infants on incubator oxygen spent more time below 85% in the current study but there was no difference in the duration of time less than 80%.

Overall, the relatively high number of episodes of hypoxemia including cerebral and abdominal hypoxemia in the current study of preterm infants on supplemental oxygen was consistent with prior studies.^1,4,22^ Devices that automatically adjust the fraction of inspired oxygen in response to changes in oxygen saturations have demonstrated improvements in oxygen saturation targeting.^10–12^ However, in spite of improvements in oxygen saturations, automated inspired oxygen control systems have not been shown to improve abdominal and cerebral oxygenation on near-infrared spectroscopy.^19^ Longer-term studies targeting a reduction in hypoxemic events would ultimately be needed to clarify if these events are causally related to adverse outcomes among preterm infants.

## Conclusion

There was no difference in episodes of intermittent hypoxemia by mode of oxygen supplementation in preterm infants with evolving bronchopulmonary dysplasia. Improvements in some measures of oxygenation may have been due to higher carbon dioxide levels during incubator oxygen treatment.

## Data Availability

Deidentified individual participant data may be requested from the corresponding author on reasonable request through a data use agreement per NIH Data Sharing Policy.
